# Aortic valve neocuspidization in children: A systematic review and meta-analysis

**DOI:** 10.1016/j.xjon.2025.101547

**Published:** 2025-12-05

**Authors:** Andrey Semyashkin, Julia Nesteruk, Lotfi Ben Mime

**Affiliations:** a Department of Pediatric Cardiac Surgery and Congenital Heart Defects, Klinikum Stuttgart, Stuttgart, Germany; b University Heart Center Freiburg–Bad Krozingen, Freiburg, Germany; c Center for Congenital Heart Defects and Pediatric Heart Center, Pediatric Heart Surgery, HDZ-NRW, University Hospital of the Ruhr University Bochum, Medical Faculty OWL University Bielefeld, Bad Oeynhausen, Germany

**Keywords:** aortic valve reconstruction, Ozaki procedure, aortic valve neocuspidization, congenital aortic valve disease, pediatric cardiac surger, autologous pericardium

## Abstract

**Background:**

Aortic valve neocuspidization (AVNeo) has emerged as a reconstructive alternative for children with aortic valve disease who are poor candidates for prosthetic replacement or the Ross procedure. Although early clinical results appear favorable, concerns persist regarding mid-term durability and material-related valve degeneration. To clarify these uncertainties, we systematically evaluated global pediatric AVNeo outcomes, focusing on early performance, mid-term reintervention rates, and the influence of pericardial material on valve longevity.

**Methods:**

A systematic search of PubMed, Embase, Scopus, the Cochrane Library, and preprint servers (January 2000-October 2025) identified studies reporting neocuspidization in patients age ≤18 years. Two reviewers independently screened and extracted data. Pooled proportions were calculated with a random-effects model; heterogeneity was assessed with the *I*^2^ statistic. Risk ratios compared reoperation rates between autologous and xenopericardial reconstructions, and comparative cohorts versus the Ross operation were summarized narratively.

**Results:**

Twelve studies including 336 children met the inclusion criteria. Early mortality was 1.2%, and late mortality was 1.1%. The pooled reoperation rate was 15.4% (95% confidence interval [CI], 5.4%-29.2%; *I*^2^ = 86%). Reoperation was 7-fold more frequent after xenopericardial reconstruction (risk ratio, 7.09; 95% CI, 2.95-17.06). Comparative series consistently favored the Ross operation for mid-term durability.

**Conclusions:**

AVNeo provides excellent early outcomes but limited mid-term durability, particularly with xenopericardium. Autologous pericardium markedly reduces reoperation risk and should remain the preferred material.

## Graphical Abstract

**Figure:**
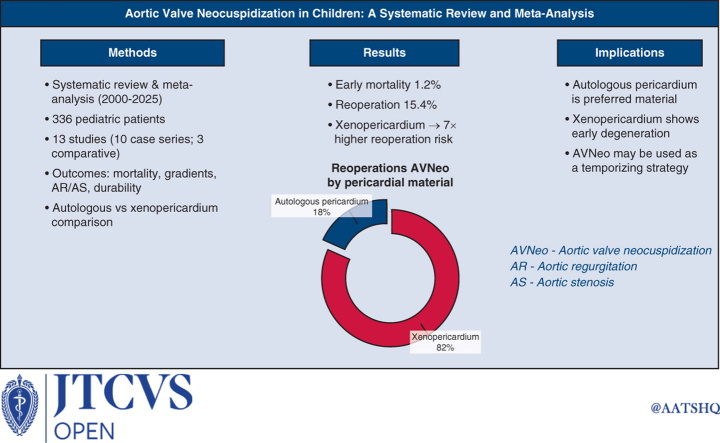

**Figure:**
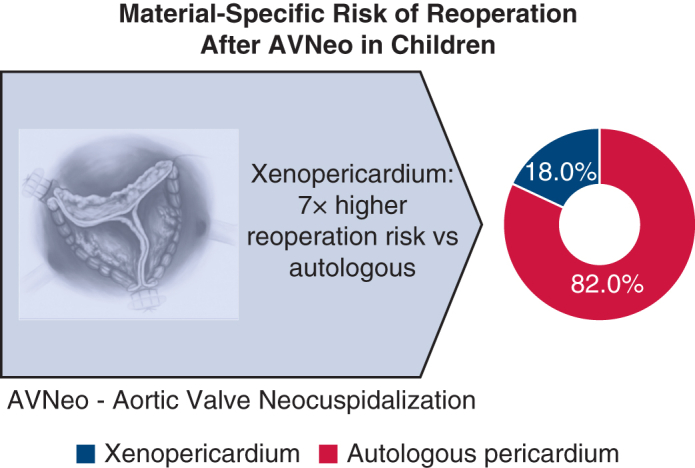
Mid-term durability of pediatric neocuspidization. Xenopericardium is associated with a markedly higher reoperation risk compared to autologous tissue.

**Central Message**

In children, aortic valve neocuspidization offers excellent early hemodynamics and low mortality, but mid-term durability is limited—especially with xenopericardium. Autologous pericardium reduces reoperation risk and is preferred.

**Perspective**

Aortic valve neocuspidization provides a useful reconstructive option in selected pediatric patients when prosthetic replacement or the Ross operation is infeasible; however, patch degeneration and reintervention risk limit durability. Careful patient selection and preference for autologous pericardium are key, and long-term data are needed to clarify indications and optimize materials.

Aortic valve disease in neonates, infants, children, and adolescents encompasses a wide spectrum of congenital and acquired abnormalities, including unicuspid, bicuspid, and quadricuspid morphology; truncal valve anomalies; and postinflammatory lesions such as rheumatic endocarditis. Conventional surgical options—balloon valvotomy, leaflet repair, prosthetic replacement, and the Ross operation—carry substantial limitations. Mechanical prostheses provide long-term durability but require lifelong anticoagulation and lack growth potential, whereas bioprosthetic valves undergo rapid calcific degeneration in young patients. The Ross operation replaces the diseased aortic valve with the pulmonary autograft and is durable in expert hands, yet it converts a single-valve disease into a double-valve reconstruction and may fail because of autograft dilatation or conduit degeneration.^1^, ^2^, ^3^ These challenges have renewed interest in reconstructive techniques that preserve the native annulus and root architecture, maintain physiologic flow dynamics, and accommodate somatic growth.

In 2011, Ozaki and colleagues^4^ introduced aortic valve neocuspidization (AVNeo), a reconstructive method in which the diseased cusps are excised and replaced by 3 pericardial leaflets tailored to the patient's annulus using dedicated templates. The technique uses glutaraldehyde-treated autologous or xenopericardial tissue. In adults, AVNeo has demonstrated excellent early hemodynamics and low operative mortality^5^^,^^6^; however, its application in children remains a matter of debate given the uncertainties surrounding long-term durability and growth potential.

Early pediatric series reported low mortality and good valve function, but later experiences documented progressive degeneration, calcification, and high reoperation rates—particularly when bovine or tissue-engineered pericardium was used.^7^, ^8^, ^9^, ^10^ With increasing adoption of the technique worldwide and emerging comparative data arguing against the Ross operation,^3^^,^^11^, ^12^, ^13^ a comprehensive evaluation of outcomes in children has become necessary. Consequently, the present systematic review and meta-analysis aimed to synthesize the global experience with pediatric AVNeo, quantify early and mid-term outcomes, and assess how pericardial material selection influences durability and the need for reoperation. Despite increasing global use of AVNeo in children, previous studies have not clarified material-specific durability, mid-term structural degeneration, or outcomes compared with the Ross procedure. This review synthesizes all available pediatric data to address these gaps.

## Methods

### Search Strategy and Selection Criteria

This systematic review followed PRISMA and MOOSE guidelines^14^^,^^15^ and was registered in the Open Science Framework. PubMed/MEDLINE, Embase, Scopus, the Cochrane Library, medRxiv, and bioRxiv were searched from January 1, 2000, to October 6, 2025. Search terms combined pediatric descriptors (child, infant, neonate, adolescent) with the intervention (Ozaki, AVNeo, “aortic valve neocuspidization,” “neo-cusp,” “aortic valve reconstruction,” “pericardial cusp,” “leaflet reconstruction”) and the condition (aortic valve) using MeSH and Emtree terms; full strategies are provided in Appendix E1. Reference lists of included articles and relevant reviews were hand-searched. Institutional Review Board approval was not required for this study because it is a systematic review and meta-analysis of previously published data. As the study involved no access to identifiable patient information, informed patient consent for publication was not required.

Eligible studies reported ≥10 pediatric patients (age ≤18 years) undergoing complete AVNeo or neoaortic valve reconstruction using autologous or xenopericardial material. Mixed adult–pediatric cohorts were included if pediatric data were extractable. Case reports, surgical descriptions without outcomes, and studies lacking pediatric data were excluded. Comparative series of AVNeo versus the Ross operation or other techniques were included for narrative synthesis but were not pooled with single-arm data.

### Data Extraction and Outcome Definitions

Two reviewers independently screened titles and abstracts, assessed full texts, and extracted data using a standardized template; disagreements were resolved by consensus. Extracted variables included study design, country, enrollment years, sample size, demographics, valve morphology, etiology, prior interventions, pericardial material and preparation (glutaraldehyde concentration and exposure time), use of Ozaki sizing templates, operative times, concomitant procedures, follow-up duration, and outcomes.

Early mortality was defined as in-hospital or 30-day death; late mortality occurred thereafter. Reoperation referred to any return to surgery for valve dysfunction (redo AVNeo, Ross conversion, or prosthetic replacement). Valve dysfunction was defined as moderate or greater aortic regurgitation (AR) or aortic stenosis (AS) depending on the study. Structural valve degeneration included calcification or leaflet failure necessitating reintervention. Event-free survival combined freedom from death, reoperation, and structural degeneration. When denominators were missing, values were derived from available data. Additional variables—including valve morphology, underlying etiology, prior aortic valve interventions, use of sizing templates, cardiopulmonary bypass and cross-clamp times, and concomitant procedures—were extracted when reported; however, fewer than 3 studies provided outcomes stratified by these variables, precluding meaningful pooled or comparative analysis.

### Risk-of-Bias Assessment

Comparative cohort studies were evaluated with the ROBINS-I tool^16^ for bias due to confounding, selection, intervention classification, deviations from intended therapy, missing data, and outcome measurement. Case series and single-arm cohorts were assessed using the Joanna Briggs Institute checklist,^17^ focusing on case definition clarity, consecutive inclusion, completeness of follow-up, and objective outcome assessment. Certainty of evidence was rated with the GRADE approach^18^ and downgraded for risk of bias, inconsistency, indirectness, imprecision, or publication bias. Preprint studies were not pooled with peer-reviewed data.

### Statistical Analysis

Descriptive statistics were summarized for each study. Pooled proportions for early and late mortality and reoperation were calculated using a random-effects model (DerSimonian-Laird)^19^ with arcsine square root transformation to stabilize variance.^20^ Between-study heterogeneity was quantified with *I*^2^ and τ^2^, and prediction intervals were calculated to estimate expected outcome ranges in future cohorts.^21^ To compare material-specific durability, aggregated reoperation counts were extracted from studies reporting outcomes by leaflet type, and Mantel-Haenszel risk ratios were computed using a log-scale random-effects model.^22^ Comparative outcomes (AVNeo vs Ross) were synthesized narratively owing to heterogeneity. Analyses were performed in R version 4.2.2 and Python version 3.11 using the meta and statsmodels packages.^23^^,^^24^

## Results

### Study Selection and Characteristics

**Figure 1:**
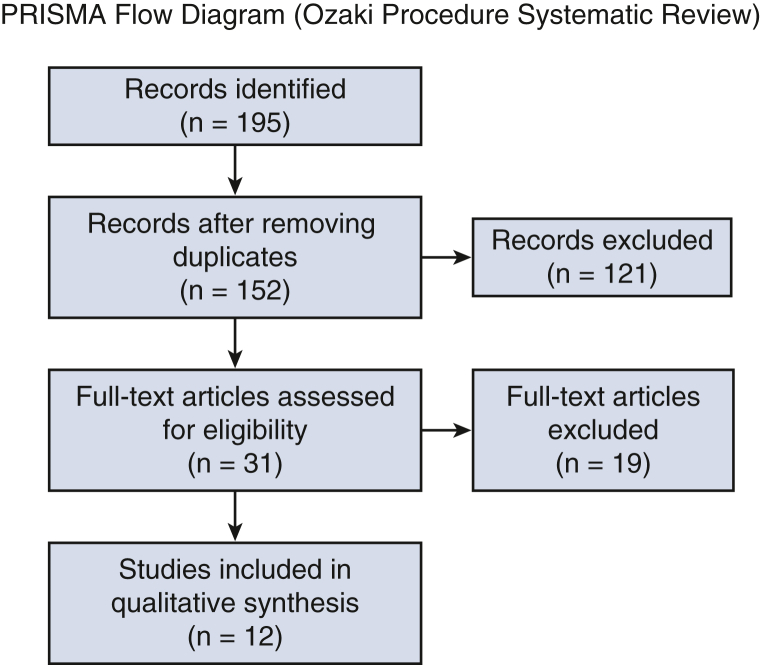
PRISMA flow diagram summarizing the study selection process.

The literature search identified 195 records, from which 43 duplicates were removed (Figure 1). After screening 152 titles and abstracts, 31 full-text articles were assessed, and 18 were excluded for adult-only cohorts or insufficient outcome data. Twelve studies met the inclusion criteria, including 10 single-arm case series or cohorts and 3 comparative studies (Table 1). Study designs included 8 retrospective case series, 2 prospective cohorts, and 2 retrospective comparative cohorts. Sample sizes ranged from 7 to 74 patients, with follow-up durations of 0.6 to 7.3 years. Reporting of valve morphology, etiology, prior interventions, sizing template use, operative times, and concomitant procedures was inconsistent, and no study provided stratified early or mid-term outcomes for these variables; therefore, no valid association analysis could be performed.

**Table 1:** Characteristics of included studies

Study	Year	Country	Study design	Sample size	Median/mean age, y	Age range	Autologous pericardium, n (%)	Xenopericar-dium, n (%)	Pericardial material	GA, %	GA time, min	FU, y, mean	Max FU, y	Early death, n	Late death, n	Reop, n	Moderate/severe AR, %	Moderate/severe AS, %
Kalezi and Majani^25^2024	2024	Tanzania	Retrospective	10	NR	>10 y (8/10)	10 (100)	0 (0)	Autologous	0.6	10	1.6	4	0	1	0	NR	NR
Polito 2021 (Pediatr Cardiol)	2021	Italy	Comparative	22	12.4	NR	NR	NR	Autologous/bovine	NR	NR	NR	NR	0	0	3	NR	NR
Huang 2023 (Transl Pediatr)	2023	China	Case series	20	7.5	IQR 5-11 y	17 (85)	3 (15)	Autologous/bovine	0.6	8	1.58	NR	0	1	3	NR	NR
Chivers 2019 (CardioCel)	2019	UK	Case series	7	NR	NR	0 (0)	7 (100)	CardioCel	NR	NR	2.47	NR	0	0	2	NR	NR
Polito 2025 (Reevaluation)	2025	Italy	Retrospective cohort	49	13.8	IQR 10.9-16.3	36 (74)	13 (26)	Autologous/xenopericardium	NR	NR	3.15	NR	0	1	11	NR	NR
Chávez 2025 (Boston)	2025	USA	Retrospective	74	12.8 ± 7.3	NR	31 (42)	43 (58)	Autologous/PhotoFix/CardioCel	NR	NR	3.2	NR	0	0	30	NR	NR
Int J Surg 2025 (China)	2025	China	Comparative	64	8.4	NR	NR	NR	NR	NR	NR	1.67	NR	0	0	5	28.1%	31.3%
EJCTS 2025	2025	International	Comparative	19	8.5	NR	NR	NR	NR	NR	NR	3.8	NR	0	NR	6	70%	NR
Mokryk 2025 (Ukraine)	2025	Ukraine	Case series	10	9	2-17 y	NR	NR	NR	NR	NR	6.08	6.08	0	0	7	NR	NR
Sivalingam 2025 (Malaysia)	2025	Malaysia	Retrospective cohort	53	10.74	NR	41 (77.4)	12 (22.6)	Autologous/CardioCel	0.6	10	2.28	7.28	1	4	4	NR	NR
Cicek 2023 (Türkiye)	2023	Türkiye	Case series	14	13.9	8.5-15 y	12 (86)	2 (14)	Autologous/bovine	NR	NR	0.71	1.58	0	0	1	NR	NR
Omar 2025 (Egypt)	2025	Egypt	Prospective cohort	25	9.1	NR	NR	NR	NR	NR	NR	1	1	0	0	0	NR	16%

*GA*, Glutaraldehyde; *FU*, follow-up; *AR*, aortic regurgitation; *AS*, aortic stenosis; *NR*, not reported; *IQR*, interquartile range.

Children undergoing AVNeo had a median or mean age between 7.5 and 13.9 years. Congenital etiologies accounted for 55% to 90% of patients in most series,^26^, ^27^, ^25^ whereas rheumatic disease predominated in the Tanzanian cohort.^28^ Valve morphology was bicuspid in up to 96% of Egyptian patients^27^ and tricuspid in the majority elsewhere. Autologous pericardium was used exclusively in several series,^28^ whereas five studies employed xenopericardial materials such as glutaraldehyde-treated bovine pericardium, PhotoFix (decellularized porcine patch), or CardioCel. Most authors used 0.6% glutaraldehyde for 10 minutes with serial saline rinses,^26^ applied Ozaki sizing templates, and avoided annular enlargement. Mean cardiopulmonary bypass time ranged from 101 ± 20 minutes to 151 ± 44 minutes, and mean cross-clamp time ranged from 87 ± 23 minutes to 125 ± 37 minutes. Concomitant procedures included mitral or tricuspid valve repair, arch reconstruction, and ventricular septal defect closure in up to 16% of patients.^27^

### Early Mortality and Hemodynamic Outcomes

Among the 336 children included in the analyzed studies, there were 4 early deaths (1.2%), including 2 (3.8%) in the Malaysian cohort,^26^ 1 (10%) in Tanzania due to severe mitral disease,^25^ and 1 (2%) in Italy from postoperative heart failure.^11^ Thus, the pooled early-mortality proportion was extremely low.

All studies reported excellent early hemodynamics; peak gradients at discharge were 9 to 16 mm Hg, mean gradients 5 to 10 mm Hg, and regurgitation was trivial or mild.^26^ Coaptation height averaged 9 to 10 mm in the Malaysian series, and annular *z*-scores improved significantly.^26^ Intraoperative conversion to a Ross or mechanical replacement occurred in only 1 patient, owing to a residual gradient.^27^ Early and late outcomes across all studies are summarized in Table 2.

**Table 2:** Summary of outcomes

Study	n	Early death, n (%)	Late death, n (%)	Reoperation, n (%)	Moderate/severe AR, %	Moderate/severe AS, %
Kalezi and Majani,^25^ 2024	10	0 (0)	1 (10)	0 (0)	NR	NR
Polito 2021 (Pediatr Cardiol)	22	0 (0)	0 (0)	3 (13.6)	NR	NR
Huang 2023 (Transl Pediatr)	20	0 (0)	1 (5)	3 (15)	NR	NR
Chivers 2019 (CardioCel)	7	0 (0)	0 (0)	2 (28.6)	NR	NR
Polito 2025 (Reevaluation)	49	0 (0)	1 (2.0)	11 (22.4)	NR	NR
Chávez 2025 (Boston)	74	0 (0)	0 (0)	30 (40.5)	NR	NR
Int J Surg 2025 (China Ozaki)	64	0 (0)	0 (0)	5 (7.8)	28.1	31.3
EJCTS 2025 (Intl cohort)	19	0 (0)	NR	6 (31.6)	70	NR
Mokryk 2025 (Ukraine)	10	0 (0)	0 (0)	7 (70)	NR	NR
Sivalingam 2025 (Malaysia)	53	1 (1.9)	4 (7.5)	4 (7.5)	NR	NR
Cicek 2023 (Turkiye)	14	0 (0)	0 (0)	1 (7.1)	NR	NR
Omar 2025 (Egypt)	25	0 (0)	0 (0)	0 (0)	NR	16

*AR*, Aortic regurgitation; *AS*, aortic stenosis; *NR*, not reported.

### Late Mortality and Valve Durability

Four late deaths occurred in the Malaysian series (7.5%), including 2 from pneumonia, 1 from septic shock after a Bentall procedure, 1 from early endocarditis.^26^ In the Tanzanian cohort, 1 death occurred at 1 year (10%).^25^ Thus, the pooled late mortality was 1.1% (95% confidence interval [CI], 0.03%-3.49%; *I*^2^ = 45%). No late deaths occurred in the Italian study or other included studies. Freedom from reoperation at 3 years ranged from 59% to 92%.

**Figure 2:**
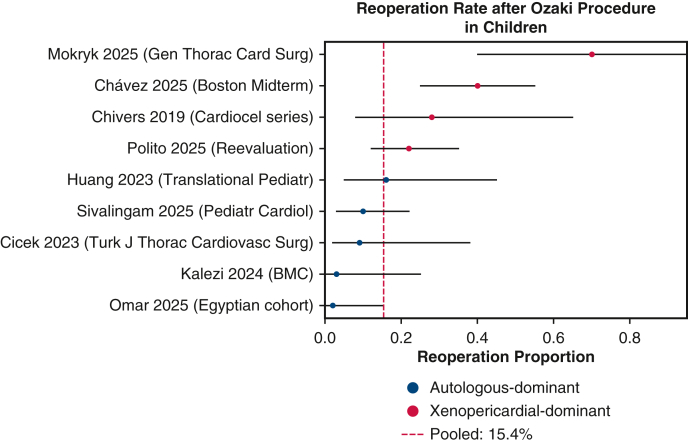
Forest plot of reoperation rates. Individual study proportions with 95% confidence intervals are shown. Blue markers represent autologous pericardium; red markers, xenopericardial material. The *dashed vertical line* denotes the pooled random-effects estimate (15.4%).

The pooled reoperation rate across 10 case series was 15.4% (95% CI, 5.4%-29.2%; *I*^2^ = 86%) (Figure 2). Median time to reintervention was 5.2 years in the Ukrainian series^13^ and 2.3 years in the Boston series.^9^ Structural degeneration manifested as cusp calcification or severe regurgitation and occurred in 7.5% of children in the Malaysian cohort. Infective endocarditis complicated 5.6% of patients, frequently necessitating root replacement. Postoperative valve gradients increased progressively over time; the median peak gradient rose from 12 mm Hg at discharge to 25 to 28 mm Hg at late follow-up in several series.^26^

### Effect of Pericardial Material

Durability differed markedly according to the pericardial material used. Tanzanian, Chinese, and Italian early-experience series using exclusively autologous pericardium reported no reoperations over 1.5 to 4 years of follow-up.^8^^,^^11^^,^^25^ In contrast, the United Kingdom and Boston cohorts using bovine or decellularized pericardium demonstrated much higher failure rates; 58% of PhotoFix reconstructions required reoperation within 3 years,^9^ and one-third of CardioCel recipients required early replacement.^7^ In the Italian early experience, all reoperations were done in patients with xenopericardium.^11^ A meta-analysis of material-specific outcomes showed that xenopericardial reconstruction increased the risk of reoperation by 7-fold compared with autologous pericardium (risk ratio 7.09; 95% CI, 2.95-17.06). These results underscore the importance of using autologous, glutaraldehyde-treated pericardium whenever possible in children undergoing AVNeo.

### Comparative Studies: AVNeo Versus Ross and Other Techniques

Three comparative studies provided detailed insights into the relative performance of AVNeo. In a single-center cohort of 117 children with complex aortic valve disease, 64 underwent AVNeo and 53 underwent the Ross procedure between 2017 and 2023.^29^ The AVNeo group had a higher prevalence of preoperative heart failure, but the 2 groups were similar in age. Preoperative extracorporeal membrane oxygenation was used exclusively in patients with an absolute or relative contraindication to the Ross procedure. During a mean follow-up of 20 months, moderate or severe aortic regurgitation developed in 28% of the AVNeo patients versus 3% in the Ross group, and moderate or severe aortic stenosis occurred in 31% versus 6%, respectively. Reoperation occurred in 7.8% of patients after AVNeo and in none of the Ross patients. The original study did not report body surface area–based analyses, and no conclusions regarding body surface area could be drawn.^29^

A multicenter European study comparing 35 Ross and 19 AVNeo procedures found a freedom from moderate or greater dysfunction at 4 years of 88% for Ross versus 30% for AVNeo, and freedom from reintervention of 94% versus 71%, respectively.^12^ These findings confirm that although AVNeo achieves satisfactory early outcomes, the Ross operation remains the reference standard for durable pediatric aortic valve replacement. Nevertheless, AVNeo may serve as a useful temporizing option in children with small annuli, complex anatomy, or contraindications to pulmonary autografting.

### Recently Published Cohorts

In a Ukrainian case series reported in 2025, 10 children (median age, 9 years), no early deaths occurred, but 7 children required reoperation at a median of 62 months, mostly for progression to mechanical or Ross replacement.^13^ The authors concluded that AVNeo is a valuable intermediate procedure but not a definitive solution.

The Boston cohort, the largest to date (n = 74; mean age 12.8 years), compared autologous and PhotoFix pericardium.^9^ Freedom from reoperation at 3 years was 86% for autologous versus 75% for PhotoFix; overall, 40% of the cohort required reoperation within 3 years, with no deaths reported.

A prospective Malaysian cohort study of 53 children using both autologous and CardioCel pericardium reported an event-free survival of 57% at 7 years, 5 late deaths, and 4 reinterventions.^26^ A Turkish congenital series of 14 patients (median age, 13.9 years) reported 1 reoperation at 8 months after bovine pericardium implantation and no deaths.^30^ An Egyptian prospective cohort of 25 patients followed for 1 year reported trivial or mild regurgitation in 80% and moderate aortic stenosis in 16%; there were no deaths, and 1 patient underwent intraoperative conversion to the Ross operation because of a residual gradient.^27^

Together, these recent series demonstrate a consistent pattern of excellent early survival and hemodynamics followed by gradual structural degeneration and increasing gradients during mid-term follow-up, especially in reconstructions using xenopericardial tissue.

### Risk of Bias and Certainty of Evidence

Most included studies were small, single-center, retrospective series without a control group. Comparative cohorts exhibited serious risk of bias owing to nonrandomized allocation, confounding, and unbalanced preoperative characteristics. Case series often lacked consecutive inclusion, standardized outcome definitions, or uniform echocardiographic follow-up.

According to ROBINS-I and the Joanna Briggs Institute checklists, comparative cohorts showed serious risk of bias from confounding and outcome assessment. The overall certainty of evidence, graded by the GRADE approach, was low to very low for all outcomes because of observational design, imprecision, inconsistency (*I*^2^ up to 86%), and suspected publication bias. Despite these limitations, the repeated observation across studies of early degeneration and high reoperation rates with xenopericardium provides moderate-strength evidence favoring glutaraldehyde-treated autologous pericardium as the material of choice for pediatric AVNeo.

## Discussion

This systematic review provides the most comprehensive synthesis to date of outcomes after AVNeo in children. It makes 3 key contributions to the pediatric AVNeo literature: it offers the first pooled, material-specific estimate showing a markedly higher reoperation risk with xenopericardium; it synthesizes all available comparative data to contextualize AVNeo relative to the Ross procedure; and it integrates temporal hemodynamic and structural findings to clarify characteristic mid-term degeneration patterns. Together, these results define current evidence boundaries and highlight the need for coordinated multicenter data collection.

Across the 12 studies including 336 patients, AVNeo was associated with very low early mortality (1.2%) and favorable early hemodynamics, with mean gradients of 5 to 10 mm Hg and peak gradients <15 mm Hg at discharge. Regurgitation was trivial or mild in nearly all cases. These early outcomes are similar to those reported for the Ross operation and generally favorable compared with prosthetic valve replacement. The technique's principal advantages—avoidance of lifelong anticoagulation, preservation of the native root architecture, and potential for annular growth—explain its growing adoption, especially in centers treating small children or operating under limited-resource conditions.

Nevertheless, mid-term durability remains limited. The pooled reoperation rate of 15% (95% CI, 5.4%-29.2%; *I*^2^ = 86%) differs from with the <5% reintervention rate typically reported after the Ross operation. Freedom from dysfunction declined to 57% at 7 years in the largest prospective cohort. Valve deterioration resulted from cusp calcification, thickening, or retraction; late infective endocarditis accounted for additional failures. Even autologous pericardium may calcify in the inflammatory milieu of congenital aortic disease.

Material choice emerged as the strongest determinant of outcome. Xenopericardial patches (ie, PhotoFix, CardioCel) exhibited early degeneration with calcification or shrinkage, yielding a reoperation rate of up to 40% to 60% reoperation. In contrast, autologous glutaraldehyde-treated pericardium showed better durability in available series. The meta-analysis demonstrated a 7-fold higher reoperation risk with xenopericardium compared with autologous tissue. The broader pathology literature confirms accelerated mineralization and collagen disruption in bovine pericardium.^31^^,^^32^ One report suggested similar outcomes with autologous pericardium and bovine pericardium,^33^ but this was based on only 4 adult patients and lacks pediatric applicability. This is consistent with our pooled evidence showing limited mid-term durability—particularly with xenopericardial tissue—and reinforces the need for careful patient selection and structured long-term surveillance. These observations align with the perspective of Konstantinov and colleagues,^34^ who emphasized that AVNeo in children behaves functionally as a biological valve replacement rather than a growth-adaptive repair, given the absence of intrinsic leaflet growth and the potential for structural degeneration over time. Comparative data reinforce more durable outcomes in available comparative cohorts of the Ross operation for long-term durability. In a 117-patient cohort, moderate or severe regurgitation developed in 28% after AVNeo but in only 3% after Ross; reoperation occurred in 7.8% versus none. Nonetheless, Ross carries its own challenges—conversion of single-valve disease to two-valve pathology, autograft dilation, and conduit degeneration. In infants and children with small annuli, endocardial infection, or contraindication to autograft transfer, AVNeo offers a potential temporizing option or bridge to definitive replacement.

Younger age and lower body surface area were consistent predictors of reoperation, implying that leaflet growth not parallel annular expansion.^26^^,^^29^ The optimal glutaraldehyde concentration and fixation duration (most commonly 0.6% for 10 minutes) remain empiric; excessive crosslinking may accelerate calcification.^31^^,^^32^ Anticoagulation practice also varies; some centers administer warfarin for 6 weeks, while others do not administer warfarin.^7^^,^^26^ Standardizing perioperative and postoperative protocols could improve outcomes.^6^^,^^11^

### Study Limitations

This meta-analysis is limited by the small number and retrospective nature of most of the included studies, heterogeneity of follow-up duration, and incomplete reporting of hemodynamic and material-specific outcomes. Publication bias cannot be excluded, as centers with early failures or limited experience might not have published their results. Comparative cohorts were nonrandomized and likely subject to selection bias, because sicker or smaller patients often underwent AVNeo instead of the Ross operation. Although several potentially relevant clinical and operative factors—such as morphology, etiology, prior interventions, sizing template use, and operative times—were extracted, the heterogeneity and incomplete reporting across studies prevented any reliable assessment of their association with early outcomes, mid-term durability, or reoperation risk. The reliance on aggregated rather than patient-level data also precluded time-to-event analyses and adjustment for confounding variables.

### Clinical Implications

AVNeo provides an attractive, resource-sparing reconstructive option in children for whom prosthetic replacement or the Ross operation is unsuitable. However, durability depends critically on the choice of material and surgical technique. Glutaraldehyde-treated autologous pericardium demonstrates markedly superior resistance to degeneration compared with xenopericardial patches and should be the preferred option. Surgeons should view AVNeo as a bridge procedure that preserves the aortic root and delays prosthetic replacement until growth is complete. Multicenter registries and standardized reporting of outcomes are needed to refine patient selection and validate technical modifications aimed at improving long-term performance.

## Conclusions

In pediatric patients with aortic valve disease, AVNeo shows low early mortality and satisfactory early hemodynamic performance, whereas mid-term durability remains limited. Across available cohorts, reoperation occurs in approximately 1 in 6 patients, with higher rates reported when xenopericardial materials are used. Across observational studies, autologous pericardium has been associated with lower reoperation rates compared with xenopericardial tissue. In comparative cohorts included in this review, the Ross operation demonstrated more durable mid-term outcomes, although its applicability depends on individual patient characteristics and institutional expertise. Current evidence suggests that AVNeo may serve as a temporary reconstructive option in selected patients when prosthetic valve replacement or the Ross procedure is infeasible. Further multicenter studies with extended follow-up are needed to clarify indications, optimize material selection, and evaluate emerging biomaterials.

## Conflict of Interest Statement

The authors reported no conflicts of interest.

The *Journal* policy requires editors and reviewers to disclose conflicts of interest and to decline handling or reviewing manuscripts for which they may have a conflict of interest. The editors and reviewers of this article have no conflicts of interest.

## Appendix E1. Search Strategy and Study Selection Methods

This appendix provides a detailed description of the literature search strategy, study selection process, and methodological framework used in this systematic review.

### Literature Search Strategy

A comprehensive and systematic literature search was conducted in accordance with the PRISMA 2020 and PRISMA-S (Search Reporting Extension) guidelines. The search was designed to identify all relevant studies reporting outcomes of aortic valve neocuspidization (AVNeo) in pediatric populations. The following electronic databases were searched: PubMed/MEDLINE, Embase (Elsevier), Scopus, and the Cochrane Library. In addition, preprint servers (medRxiv and bioRxiv) were searched to capture relevant grey literature. The search covered publications from January 1, 2000, to October 6, 2025 (Europe/Berlin time zone). No language or study design restrictions were applied. Human studies were included when applicable.

Search strategies combined controlled vocabulary terms (Medical Subject Headings [MeSH] and Emtree terms) and free-text keywords related to the population, intervention, and condition of interest. Pediatric population terms included *infant, neonate, child, pediatric/paediatric*, and *adolescent*. Intervention-related terms included *Ozaki, AVNeo, aortic valve neocuspidization, neo-cusp, aortic valve reconstruction, pericardial cusp*, and *leaflet reconstruction*. These terms were combined with descriptors related to the aortic valve using Boolean operators. Full electronic search strategies for each database are available on request.

### Study Selection Process

All records identified through the search were imported into a reference management system, and duplicate records were removed prior to screening. Titles and abstracts were screened independently to exclude clearly irrelevant studies. Full-text articles subsequently were retrieved for all potentially eligible records and assessed for inclusion based on predefined eligibility criteria.

Studies were eligible for inclusion if they reported clinical outcomes of AVNeo in pediatric or adolescent patients. Case series, cohort studies, and comparative studies were considered eligible. Studies were excluded if they involved exclusively adult populations, did not report original clinical data, focused on nonsurgical interventions, or lacked extractable outcome data relevant to the review objectives.

Disagreements during study selection were resolved by consensus.

### Data Extraction and Classification

For each included study, data were extracted on study design, patient demographics, surgical technique, pericardial material, follow-up duration, and clinical outcomes, including mortality, reoperation, and valve-related complications. Studies were classified according to design (retrospective case series, prospective cohort studies, and retrospective comparative cohorts) for descriptive and analytical purposes.

### Methodological Consistency

The study selection and data extraction processes were conducted to ensure methodological transparency, minimize bias, and prevent duplicate data representation. The final dataset formed the basis for qualitative synthesis and quantitative analyses reported in the main manuscript.

## References

[1] Etnel J.R.G., Takkenberg J.J.M., Elmont L.C. (2018). The Ross procedure in children: a systematic review and meta-analysis. J Am Coll Cardiol.

[2] Etnel J.R.G., Huygens S.A., Grashuis P. (2023). Ross procedure in children: updated long-term outcomes. Eur J Cardiothorac Surg.

[3] Polito A., Moya M., Trezzi M. (2021). Aortic valve neocuspidization versus Ross operation in children: a propensity-matched study. Pediatr Cardiol.

[4] Ozaki S., Kawase I., Yamashita H. (2011). Aortic valve reconstruction using self-developed templates for aortic valve disease. J Thorac Cardiovasc Surg.

[5] Ozaki S., Kawase I., Yamashita H. (2018). Aortic valve reconstruction using autologous pericardium: 12-year follow-up. J Thorac Cardiovasc Surg.

[6] Shrestha M., Folliguet T., Pfeiffer S. (2016). Aortic valve neocuspidization with autologous pericardium: early results of the first 100 patients. Eur J Cardiothorac Surg.

[7] Chivers L., Tsang V., Brawn W.J. (2019). Ozaki procedure with CardioCel patch in children and young adults: preliminary experience—a word of caution. World J Pediatr Congenit Heart Surg.

[8] Huang J., Zhao X., Gao Y. (2023). Midterm follow-up of aortic valve neocuspidization with pericardium in children. Transl Pediatr.

[9] Chávez A.M., d’Udekem Y., Alsoufi B. (2025). Mid-term results of Ozaki technique for aortic valve reconstruction: autologous versus PhotoFix pericardium in congenital aortic and truncal valve disease. JTCVS Struct Heart Endovasc.

[10] Shibata T., Ikeda T., Matsumoto H. (2023). Histologic changes of neocusps after Ozaki procedure in children. Ann Thorac Cardiovasc Surg.

[11] Polito A., Di Filippo S., De Rita F. (2025). Reevaluating aortic valve neocuspidization in children, adolescents, and young adults. World J Pediatr Congenit Heart Surg.

[12] Zhang W., Jiang Q., Liu Y. (2025). Surgical outcomes of aortic valve replacement in children with Ross and Ozaki procedure. Eur J Cardiothorac Surg.

[13] Mokryk I., Dzyadzko V., Milyutina E. (2025). Neocuspidization in congenital aortic valve disease: mid-term results in children. Gen Thorac Cardiovasc Surg.

[14] Page M.J., McKenzie J.E., Bossuyt P.M. (2021). PRISMA 2020 statement: an updated guideline for reporting systematic reviews. BMJ.

[15] Stroup D.F., Berlin J.A., Morton S.C. (2000). Meta-analysis of observational studies in epidemiology (MOOSE). JAMA.

[16] Sterne J.A.C., Hernán M.A., Reeves B.C. (2016). ROBINS-I: a tool for assessing risk of bias in non-randomized studies. BMJ.

[17] Moola S., Munn Z., Sears K. (2020). JBI manual for evidence synthesis, chapter 7: systematic reviews of etiology and risk. https://jbi-global-wiki.refined.site/space/MANUAL/355598596/7.+Systematic+reviews+of+etiology+and+risk.

[18] Guyatt G.H., Oxman A.D., Vist G.E. (2008). GRADE: an emerging consensus. BMJ.

[19] DerSimonian R., Laird N. (1986). Meta-analysis in clinical trials. Control Clin Trials.

[20] Freeman M.F., Tukey J.W. (1950). Transformations related to the angular and the square root. Biometrics.

[21] Higgins J.P.T., Thompson S.G., Spiegelhalter D.J. (2009). Re-evaluation of random-effects meta-analysis. J R Stat Soc Ser A.

[22] Mantel N., Haenszel W. (1959). Statistical aspects of retrospective data. J Natl Cancer Inst.

[23] R Core Team (2022).

[24] Seabold S., Perktold J. (2010). Proc 9th Python in Science Conf.; 28 June-3 July, 2010; Austin.

[25] Kalezi V., Majani N. (2024). Outcomes post-Ozaki procedure among children with aortic valve disease. BMC Cardiovasc Disord.

[26] Sivalingam S., Santhosh S., Lingam M. (Published online March 5, 2025). Factors predicting early outcome of aortic valve neocuspidization in the pediatric population. Pediatr Cardiol.

[27] Omar M.E., Abdelrahman A., Elshazly M. (2025). Egyptian experience with aortic valve repair using the Ozaki procedure in adult and pediatric patients. J Cardiothorac Surg.

[28] Naik R., Raj R., Singh H. (2025). Early outcomes of aortic valve neocuspidization: initial experience of a single centre. Indian Heart J.

[29] Wang G.-X., Zhang S., Ma K. (2025). Ozaki technique versus Ross operation for complex aortic valve diseases in children. Int J Surg.

[30] Cicek M., Ucar T., Kafali H.C. (2023). Aortic valve neocuspidization in congenital aortic valve disease: early results. Turk Gogus Kalp Damar Cerrahisi Derg.

[31] Schoen F.J., Levy R.J. (2005). Calcification of tissue heart valve substitutes: progress toward understanding and prevention. Ann Thorac Surg.

[32] Sénage T., Gillaizeau F., Le Tourneau T. (2019). Structural valve deterioration of bioprosthetic aortic valves: an underestimated complication. J Thorac Cardiovasc Surg.

[33] Jubouri M., Tan S.Z.C.P., Mohammed I., Bashir M. (2022). Aortic valve neocuspidization using autologous versus bovine pericardium: Ozaki versus Batista. J Card Surg.

[34] Konstantinov I.E. (2021). Commentary: ozaki valve reconstruction in children: is it still a valve replacement?. J Thorac Cardiovasc Surg.

